# Assessment of Cytotoxicity, Fetotoxicity, and Teratogenicity of *Plathymenia reticulata* Benth Barks Aqueous Extract

**DOI:** 10.1155/2013/128594

**Published:** 2013-12-22

**Authors:** Lia de Barros Leite Albuquerque, Cháriston André Dal Belo, Marcio Galdino dos Santos, Patricia Santos Lopes, Marli Gerenutti, Yoko Oshima-Franco

**Affiliations:** ^1^Post-Graduate Program in Pharmaceutical Sciences, University of Sorocaba, UNISO, Rodovia Raposo Tavares, km 92.5, 18023-000 Sorocaba, SP, Brazil; ^2^Federal University of Pampa, CIPBiotec, UNIPAMPA, Avenida Antonio Trilha 1847, 97300-000 São Gabriel, RS, Brazil; ^3^Post-Graduate Course in Environmental Sciences, PGCiamb, Federal University of Tocantins, UFT, Avenida NS 15 ALC NO 14, 109 Norte, 77001-090 Tocantins, Brazil; ^4^Federal University of São Paulo, UNIFESP, R. Prof. Artur Riedel 275, 09972-270 Diadema, SP, Brazil

## Abstract

Scientific assessment of harmful interactions of chemicals over the entire reproductive cycle are divided into three segments based on the period: from premating and mating to implantation (I), from implantation to major organogenesis (II), and late pregnancy and postnatal development (III). We combined the segments I and II to assess *Plathymenia reticulata* aqueous extract safety. In order to investigate reproductive toxicity (segment I), pregnant rats received orally 0.5 or 1.0 g/kg of extract, daily, during 18 days. These concentrations were determined by a preliminary *in vitro* LD50 test in CHO-k1 cells. A control group received deionized water. The offspring was removed at the 19th day, by caesarean, and a teratology study (segment II) was carried out. The corpora lutea, implants, resorptions, live, and dead fetuses were then counted. Placenta and fetuses were weighted. External and visceral morphology were provided by the fixation of fetuses in Bouin, whereas skeletal analysis was carried out on the diaphanizated ones. The increase in the weights of placenta and fetuses was the only abnormality observed. Since there was no sign of alteration on reproduction parameters at our experimental conditions, we conclude that *P. reticulata* aqueous extract is safe at 0.5 to 1.0 g/kg and is not considered teratogenic.

## 1. Introduction

*Plathymenia reticulata *Benth (Leguminosae) is a plant popularly known as “vinhático” (wine-like), found in “Cerrado” region in Brazil, and represents a good source of high quality wood. The plant coevolution with other species has led to the development of secondary metabolites for its self-defenses against pathogens (viruses, bacteria, and fungi) and predators like insects and mammals.

Among several secondary metabolites identified in *P. reticulata* trunk heartwood, plathyterpol [1], vinhaticyl acetate, and methyl vinhaticoate [2], 16,18-diacetoxycass-13(15)-ene and 16-hidroxy-18-acetoxycass-13(15)-ene [3] are the most common. The medicinal potential of *P. reticulata* as anti-inflammatory [4], antimicrobial [5, 6], and depurative of blood [7] is also highlighted by the inherent presence of tannins, flavonoids [5], and cassane diterpenes in its constitution [3].

However, in spite of their medicinal potential, secondary metabolites synthesized by plants can also be harmful for the aggressor. In mammals, they can induce toxicity to a number of different organs including skin, lung, liver, kidney and bladder, blood, skeletal muscle, and central and peripheral nervous systems including the neuromuscular junctions [8].

*In vitro* studies showed that *P. reticulata* barks hydroalcoholic extract inhibits the irreversible neuromuscular blockade induced by *Bothrops jararacussu* (79.3 ± 7.5%) and *Crotalus durissus terrificus* (73.2 ± 6.7%) venoms, on mouse neuromuscular junctions. This antivenom activity was mainly related to protein precipitation caused by the high content of tannins (4%) present in the extract [9].

Using mouse skeletal muscles, further investigations upon the anti-snake venom profile of *P. reticulata* barks secondary metabolites showed that the dichloromethane extract (0.4 mg/mL) inhibited the throphic muscle effects induced by *Bothrops jararacussu* venom [10].

In an attempt of investigating the risk assessment of *P. reticulata*, its mutagenic potential was evaluated by the *Salmonella* mutagenicity assay (Ames test) and the micronucleus test in CHO-K1 cells. Although the hydroalcoholic extract of *P. reticulata* barks showed mutagenicity, the Ames test also unveiled its anticarcinogenic potential [11].

In Brazil, local markets frequently sell herbal medicinal plants, in which tannin-rich trees, like *P. reticulata*, are commonly found. Plants rich in tannins are also described for treating diarrhea, hypertension, wounds, burns, kidney and stomach diseases, and inflammation [12]. However, in spite of their obvious clinical benefits, the oral administration of these remedies, associated with the lack of scientific proof of safety, put in risk the population health. Besides the potential adverse effects caused by the herb itself, teratogenicity is another important concern.

During pregnancy, one of the results of acute or chronic exposure to naturally occurring chemical agents can be an abnormal offspring development. Manifestations of the developmental toxicity include structural malformations, growth retardation, functional impairment, and/or death of the organism [13].

In this work we showed the safety evaluation of *P. reticulata* aqueous extract using developmental and reproductive toxicology protocols (segments I and II).

## 2. Materials and Methods

### 2.1. Vegetal Material and Extraction Procedure

Samples of* P. reticulata *Benth bark were collected from Miracema city herbarium (Miracema, Tocantins, Brazil) in December 2007. The specimens were deposited (protocols NRHTO 3327) at the herbarium of Federal University of Tocantins. The bark was dried at 40°C in an incubator with forced air circulation apparatus for 48 hours. The material was ground in a mill (MA 340, Marconi, Brazil), macerated for seven days (1276.32 g) in 70% ethanol (14.5 liters), and the suspension was protected from light and percolated at 20 drops/minute, resulting in a 20% (w/v) hydroalcoholic extract. This procedure was previously described by Farrapo et al. [10] and Della Torre et al. [11]. Briefly, the obtained extract contained 3.75% polyphenols and 0.16% flavonoids and showed positive reactions to tannins. The resulting material was concentrated in a rotary evaporator (TE-210, Tecnal, Brazil) and lyophilized (Multitasking Freeze Drying S, SNL216V-115, Thermo Fisher Scientific, USA).

### 2.2. Cell Line and Culture Conditions

As described by Della Torre et al. [11], Chinese hamster ovary cells (CHO-K1 lineage, American Type Cell Culture, ATCC number CCL-61) were maintained at 37°C in 5% CO_2_ and 97% humidity in RPMI 1640 culture medium (Gibco, USA), supplemented with 10% (v/v) fetal bovine serum (FBS, Gibco), 1% (v/v) L-glutamine (L-Glu, Gibco), 1% (v/v) penicillin streptomycin (PS, Gibco) and 0.1% (v/v) amphotericin B (Gibco). For subculturing and experiments, the cells were harvested using 0.05% (w/v) trypsin and 0.02% (w/v) ethylene diamine tetracetic acid (EDTA) in a saline phosphate-buffered solution, pH 7.4. Each trypsinization was recorded as one passage. The test was performed at the third passage.

### 2.3. Cytotoxicity Evaluation (IC10 and IC50)

The cytotoxicity evaluation was carried out by using the CellTiter 96 AQueous Non-radioactive Cell Proliferation Assay (Promega, Madison, WI, USA), in which 3-(4,5-dimethylthiazol-2-yl)-5-(3-carboxymethoxyphenyl)-2-(4-sulphophenyl)-2H-tetrazolium, inner salt (MTS) is bioreduced to formazan by dehydrogenase enzymes in metabolically active cells. The amount of formazan produced by the cells was determined by measuring sample absorbance at 490 nm with a spectrophotometer SpectraMax 190 (Molecular Devices, São Paulo, SP, Brazil). Statistical analysis of data was performed by using one-way analysis of variance (ANOVA) between two different sample curves and solvent control curve. The binomial proportion confidence interval was adopted.

### 2.4. *In Vitro* LD50 of *P. reticulata* Barks Lyophilized Extract

The value of the LD50 (Lethal Dose to kill 50% of animals), an essential test for the controlled use of animals of assays *in vivo*, was determined in *P. reticulata* barks lyophilized extract. In this assay, the inhibitory concentration that kills 50% of the cells (IC 50), was (0.331 mg mL^−1^), as describe by Della Torre et al. [15]. Applying the formula: log [LD50 (mg mL^−1^)] = 0.372  × log IC50 (*μ*g mL^−1^) +2.024 [16, 17], the value of the LD50 was calculated in order to orientate the *in vivo* experimental assays.

### 2.5. *In Vivo* Experiment

#### 2.5.1. *P. reticulata* Aqueous Extract Preparation

The *P. reticulata* aqueous extract, to be administered via gavage in rats, was prepared daily using the previous lyophilized extract (see plant material and extraction) dissolved in deionized water.

#### 2.5.2. Animals

Six males and fifteen female adult Wistar rats weighing 160 g to 200 g were supplied by Anilab, Animais de Laboratório (Paulínia, São Paulo, Brazil). All animals were maintained in groups (5 rats per cage), previously housed to laboratory conditions during one week before the experiments at 25 ± 3°C on a 12 h light/dark cycle and had access to food and water *ad libitum*. This project (protocol number A011/CEP/2008) was approved by the institutional Committee for Ethics in Research of Vale do Paraiba University (UNIVAP), and the experiments were carried out according to the guidelines of the Brazilian College for Animal Experimentation.

#### 2.5.3. The Reproduction and Fertility Study (Segment I)

The method for reproductive evaluation was previously described elsewhere [18–20]. Briefly, 15 sexually naive rat females were mated with males (five females with two males per cage). Pregnancy was confirmed through the presence of spermatozoids in vaginal-washing rubbing observed by microscopy analysis [21]. The presence of spermatozoids was considered as the first day of pregnancy. Each pregnant female was kept in separate cage. Three experimental groups were analyzed, two treated and one control. The animals had free access to water and food during all the experiment and the consumption was monitored daily. For reproductive evaluation, each group of 5 females received 0.5 g/kg/day (group 1) or 1.0 g/kg/day (group 2) of *P. reticulata* extract or deionized water (group 3, control), from the first to the 18° day of pregnancy. The weight gain of pregnant females was monitored during the pregnancy.

#### 2.5.4. The Teratology Study (Segment II)

For the teratogenic study each group of 5 females received by gavage 0.5 g/kg/day (group 1), 1.0 g/kg/day (group 2) of *P. reticulata* extract, or deionized water (group 3) from days 1 to 18 of pregnancy. Pregnant rats were anesthetized with halothane (Halotano, Cristalia, Brazil), killed, and submitted to a rapidly excision of their uterus. The following macroscopic parameters were evaluated in order to observe the reproductive performance of rats [22]: (1) placenta weight (grams); (2) fetus weight (grams); (3) preimplantation loss (%) = corpora lutea number − implantation number/corpora lutea number; (4) postimplantation loss (%) = implantation number − alive fetus/implantation number; and (5) offspring vitality (%).

The offspring was anesthetized with halothane, killed, and fixed in Bouin's solution for 24–48 h, replaced by 70% hydroalcoholic solution. The following parameters were measured (cm): A: craniocaudal; B: tail; C: anteroposterior of cranio; D: laterolateral of cranio; E: anteroposterior of thorax; and F: laterolateral of thorax. The other offspring group was anesthetized with halothane, killed, eviscerated, and diaphanizated for posterior skeletal examination. The fetuses selected were fixed in ethanol, then “cleared” and stained by a KOH alizarin red-S technique [23]. Examination included enumeration of the vertebra, ribs, and other bone structures, degree of ossification, and any fusions or abnormalities in bone shape or position [24].

### 2.6. Statistical Analysis

Data from the different assays were first analyzed regarding distribution and variance homogeneity. Normally distributed data were submitted to comparison between both groups by using Student's *t*-test. Nonnormally distributed data were first transformed (log). One-way ANOVA or Fisher's exact tests were used for evaluation of physical development parameters. Significance level was set as 5%.

## 3. Results and Discussion

This study was designed to evaluate the safety of oral administration of *P. reticulata* aqueous extract in pregnant rats.

A prerequisite for understanding the abnormal development in mammals is the evaluation of the normal development that, in turn, is characterized by changes such as size, biochemistry and physiology, and in shape and functionality. In this view, gametogenesis is the process of forming the haploid germ cells, the egg, and sperm. These gametes fuse in the process of fertilization to form the diploid zygote,the embryo. It is well known that, in developmental toxicity studies, the major effects of prenatal exposure of a chemical compound are observed at the time of birth as embryolethality, malformations, and growth retardation. A disturbance on a single cell may induce an abnormal development at the zygote (one-cell) stage, the blastocyst stage (when only a few cells in the inner cell mass are embryo progenitors), or during organogenesis, when organ rudiments may consist of only few cells. Nevertheless, the relationship between these effects is too complex to evaluate and varies with the type of agent, the time of exposure, and the dose of the toxic compound [13].

**Figure 1:**
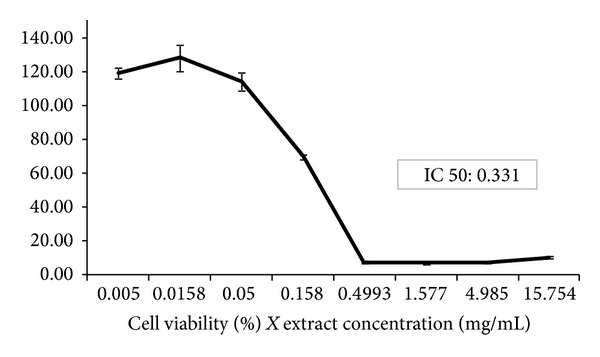
Effect of *P. reticulata* hydroalcoholic extract against Chinese hamster ovary cell viability (%). The graph shows the cell viability versus different concentrations of *P. reticulata* extract (mg/mL). The IC10 value was estimated in 0.113 mg/mL and the IC50 in 0.331 mg/mL, calculated via Phototox software program [14].

Figure 1 shows the cell viability (%) compared to different plant extract concentrations. The value of IC10 was found to be 0.113 mg/mL, accounting for the concentration at which approximately 90% of cells survived (noncytotoxic concentration). The IC50 of 0.331 mg/mL corresponds to the concentration at which approximately 50% of the cells survived.

De Toledo et al. [6] evaluated *Plathymenia reticulata* cytotoxicity using VERO cells and found a cytotoxic concentration (CC50) of 156.67 *μ*g/mL. A possible explanation for these different values may relay in the different cell line used and also the different colorimetric assays.

According to ICCVAM [16], the LD50 value can be determined based on the IC50, by applying the following formula: log LD50 (mg/kg) = 0.372 log IC50 (*μ*g/mL) + 2.024. Thus, the advantages of using this analysis are the reduction of animal use during *in vivo* tests [14], and the knowledge of the initial dose for *in vivo *studies, mainly when LD50 tests for acute oral toxicity, is required. Nowadays, most of the toxicological studies involving new drugs combine *in vivo *and *in vitro *assays in order to increase safety (i.e., in the case of a further approval for clinical use). For example, the evaluation of the development and safety of medicinal products require the estimation of IC50 values [17]. In our experimental conditions, together with the IC50 values in CHO-K1 cells, it was calculated the LD50 as 915 mg/kg. The above calculations applied to the *in vivo* experimental assays permitted the determination of two concentrations (0.5 and 1 g/kg) of *P. reticulata* extract, that mimics the human consumption.

**Figure 2:**
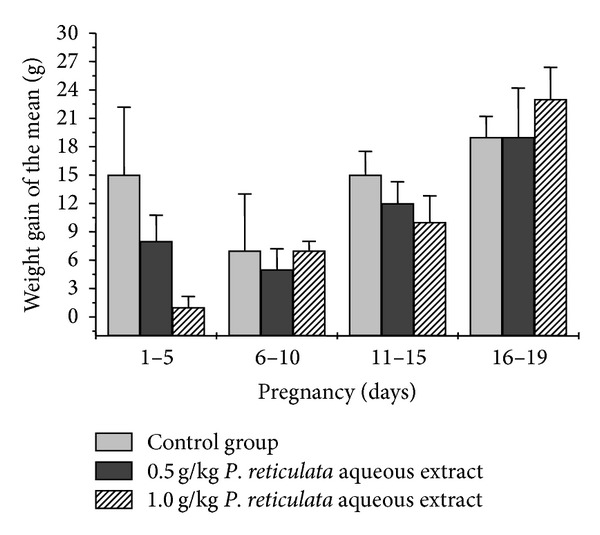
Effect of *P. reticulata* aqueous extract on weight gain of pregnant rats. On the graph each bar is the mean ± S.E.M. of five experiments. Note that there were no significant changes (*P* > 0.05, *t*-test, and one-way ANOVA test were applied in this assay) between the *P. reticulata*-treated and control groups.

Figure 2 shows the graph of mean (±S.E.M.) weight gain during the gestation, considering water ingestion and food consumption *ad libitum*. At the 95% confidence level the two means (control compared to *P. reticulata extracts*) are not significantly different. The weight losses of control pregnant rats and treated groups and also of 6–10th day of pregnancy compared to 1–5th day can be explained by the habituation phenomenon [25], since rats are very sensitive to manipulation.

The relationship between maternal and developmental toxicity is not only a result of an insult to the conceptus at the cellular level. Insults may occur through different routes, including a direct effect on the embryo/fetus, indirect toxicity of the agent to the mother through the placenta, or a combination of direct and indirect effects. Maternal conditions could potentially harm the developing organism by altering the nutritional status, among several different factors [26, 27].

**Figure 3:**
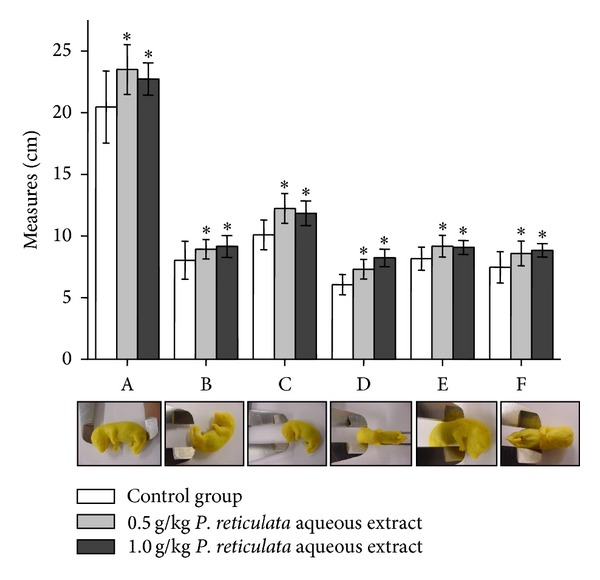
Effect of *P. reticulata* aqueous extract (0.5 g/kg and 1.0 g/kg) against the external morphological parameters of offspring. The graph is the mean ± S.E.M. of five experiments. (A: craniocaudal; B: tail; C: anteroposterior of cranio; D: laterolateral of cranio; E: anteroposterior of thorax; and F: laterolateral of thorax.) *P < 0.05 compared to control group.

It is well known that intergenerational malnutrition is responsible for reducing the gain of weight during pregnancy in rats [28]. Therefore, the distinction between direct and indirect developmental toxicity is important to understand safety assessment tests in pregnant animals. In our experimental conditions, all animals had access to food and water *ad libitum*, in order to exclude this variable (Figure 3). Here, the concentrations assayed did not induce maternal toxicity. According to Rogers and Kavlock [13], a decrease in food or water intake would induce weight loss and other clinical signs. As an example, they have shown a significant maternal weight reduction at the end of pregnancy in the sibutramine nonoverweight drug-treated group, compared to the control (nonoverweight, no drug). This data can be linked to a significant increase in post-implantation loss and placental index, suggesting that sibutramine alone or the condition of excess weight in the absence of drugs has altered the reproductive performance [15].

The reproductive performance can be also evaluated by macroscopic parameters such as placenta weight (grams), fetus weight (grams), preimplantation loss (%), postimplantation loss, and offspring vitality (%). Regarding this later information, the data after administration of *P. reticulata* aqueous extract (0.5 g/kg or 1 g/kg) did not differ from control group (Table 1), except for placenta and fetuses weights. Under these parameters, *P. reticulata* treatment increased the gain (in grams) of placenta and fetuses. According to Langley and Jackson [29], low-protein intake induces intrauterine protein restriction during diet that could explain the gain (in grams) of placenta and fetuses in *P. reticulata* treatment. However, in our experimental conditions animals had access to food and water *ad libitum*. According to des Robert et al. [30], high protein intake via the enteral route could explain the enhanced weight gain in *P. reticulata* treatment.

The external morphological parameters of offspring were measured and compared to control group. All parameters evaluated were statistically different to the control, but not between the experimental treated-groups, via mother, that received 0.5 g/kg or 1.0 g/kg of *P. reticulata* aqueous extract (Figure 3).

No abnormality was seen in fetuses, except with the offspring sizes, demonstrating the safety of *P. reticulata* aqueous extract, in our experimental conditions. When cyclophosphamide (40 mg/kg), a well-known teratogenic agent, was used as a positive control, a strong teratogenic activity was observed (a dose 12.5 and 25 times lower than 0.5 and 1.0 g/kg *P. reticulata*, resp.). At this concentration cyclophosphamide was able to induce high resorption rate (approximately, 80%) and severe fetal malformations with retarded growth. These later phenomena were traduced by craniofacial alterations such as severe microcephaly, agnathia, open eyes, limb reduction, and trunk anomalies such as phocomelia or amelia, as well as, eventration of the abdominal wall and absence of the tail. Even at 15 mg/kg of cyclophosphamide, the teratogenic effect was observed in 70% of fetuses, which exhibited external tail and digit anomalies, such as short or crooked tail, syndactyly and ectrodactyly [31].

**Figure 4:**
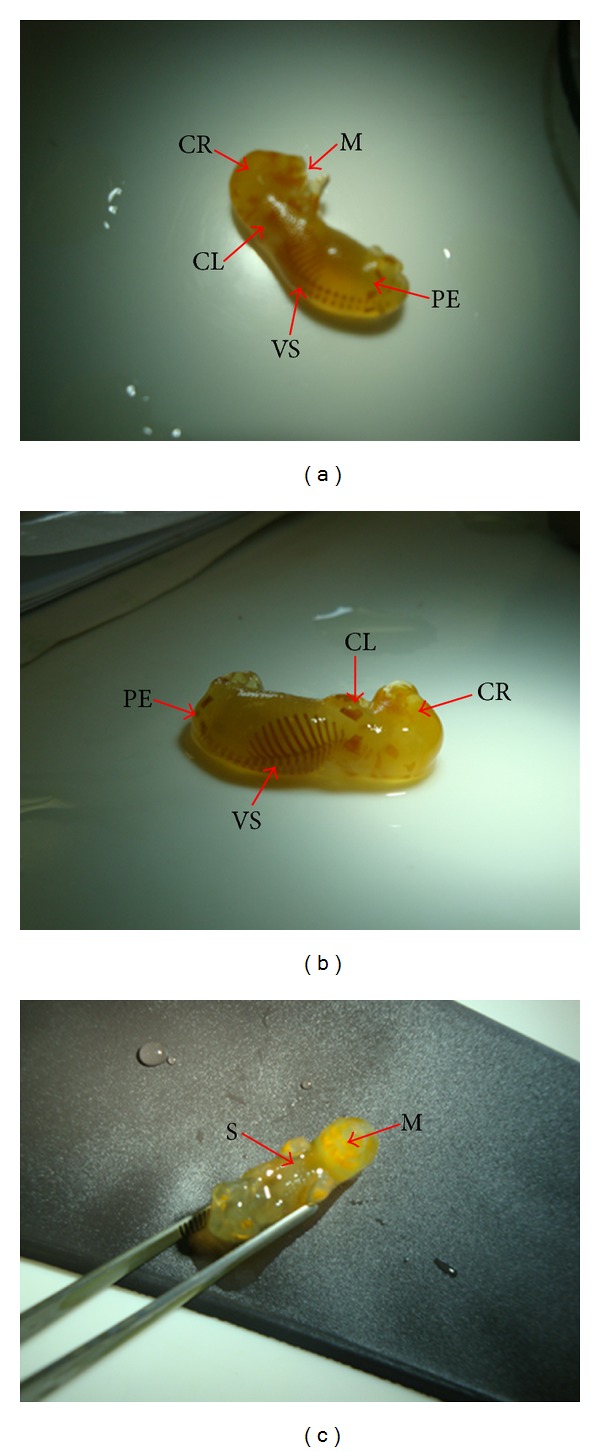
Representative pictures of 19th days gestation fetuses for teratogenicity test. Pregnant rats were treated daily with *P. reticulata* (1.0 g/kg) and the offspring removed surgically prior to birth. Diaphanizated fetuses were analyzed by lateral (a), posterior (b), and frontal (c) views. The parameters of sternum ossification (S), clavicule (CL), cranio (CR), pelvis (PE), mandible (M), and vertebral spine (VS) were examined. Notice that no abnormality was observed.

The analysis of external morphology carried out in conceptus exposed to* P. reticulata* aqueous extract (0.5 g/kg or 1/0 g/kg) just prior to birth showed no abnormalities upon skeletal examination of diaphanizated fetuses (Figure 4). The parameters analyzed were soft tissues, cartilage calcification, vertebra and ribs quantification, bone shape or position, sternum ossification (S), and bones such as cranium (CR), pelvis (PE), vertebral spine (VS), clavicule (CL), and mandible (M).

**Figure 5:**
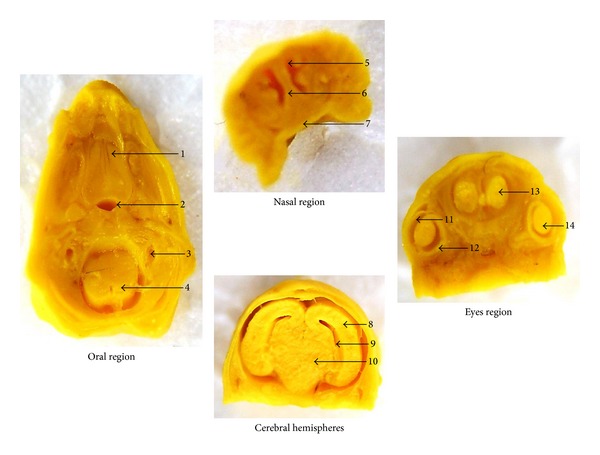
Representative sections from head and neck regions of fetuses exposed to *P. reticulata* aqueous extract (1.0 g/kg). Oral region transversally sectioned: 1: palate; 2: trachea; 3: inner ear; 4: marrow. Nasal region frontally sectioned: 5: nasal cavity; 6: nasal septum; 7: palate. Cerebral hemispheres region frontally sectioned: 8: cerebral hemisphere; 9: ventricles; 10: diencephalon. Eyes region frontally sectioned: 11: cornea; 12: retina; 13: olfactory bulb; 14: crystalline. After a careful analysis of the anatomical parameters, no abnormality was observed among the groups.

Visceral malformations were not observed in organs such as liver, stomach, duodene, and kidneys. Morphological analysis in the head and neck regions showed that all structures were correctly implanted. The observation of structures such as palate, nostrils, ocular globe, inner ear, cortex, cerebral ventricle, marrow, trachea, and esophagus showed no sign of alterations; the oral cavity was delimited by palate and was not obstructed in all fetuses examined, treated groups, and control group (Figure 5).

This study also included the daily observation of pregnant rats to a previous oral administration of *P. reticulata* aqueous extract (0.5 g/kg or 1.0 g/kg). In this protocol there were no signs of increased hair loss, excessive salivation, alteration in respiration, and abnormal gaits, tremors, or seizures. Although the gain of weight (grams) of pregnant rats was not statistically different (treated groups compared to the control group), there were significative changes in the weights of placenta and fetuses (*n* = S.E.M., *P < 0.05). The fetal evaluation between treated and control groups showed no malformations, defined as “those structural anomalies that alter general body conformity, disrupt or interfere with body function, or are generally thought to be incompatible with life” [32].

## 4. Conclusions

Overall the results suggest that oral administration of *P. reticulata* aqueous extract at 0.5 and 1.0 g/kg is safe, related to reproduction and fertility parameters or even in terms of inducing teratogenicity. This paper also shows that the combination of *in vitro* assays to select the dosage, with *in vivo* experiments, which involved the segment I (the period of premating and mating to implantation) and segment II (the period from implantation to major organogenesis), can be useful for assessing safety parameters of new medicinal compounds.

## Figures and Tables

**Table 1:** Reproductive performance of pregnant rats exposed to *Plathymenia  reticulata* aqueous extract.

Teratogenicity parameters	Control	Experimental 0.5 g/kg	Experimental 1.0 g/kg
Preimplantation loss (%)	0	0	0
Postimplantation loss (%)	0	1.69	5.55
Placenta weight (grams)	0.494 ± 0.07 (*n* = 59)	0.542 ± 0.09* (*n* = 58)	0.530 ± 0.07* (*n* = 51)
Fetus weight (grams)	1.336 ± 0.25 (*n* = 59)	1.433 ± 0.20* (*n* = 58)	1.456 ± 0.15* (*n* = 51)

*P < 0.05.
